# Vancomycin-nanofunctionalized peptide-enriched silk fibroin to prevent methicillin-resistant *Staphylococcus epidermidis*-induced femoral nonunions in rats

**DOI:** 10.3389/fcimb.2022.1056912

**Published:** 2023-01-05

**Authors:** Marta Bottagisio, Silvia Palombella, Silvia Lopa, Fabio Sangalli, Paolo Savadori, Marco Biagiotti, Zili Sideratou, Dimitris Tsiourvas, Arianna B. Lovati

**Affiliations:** ^1^ IRCCS Istituto Ortopedico Galeazzi, Laboratory of Clinical Chemistry and Microbiology, Milan, Italy; ^2^ IRCCS Istituto Ortopedico Galeazzi, Cell and Tissue Engineering Laboratory, Milan, Italy; ^3^ IRCCS Istituto di Ricerche Farmacologiche Mario Negri, Laboratory of Renal Biophysics, Department of Biomedical Engineering, Bergamo, Italy; ^4^ IRCCS Istituto Ortopedico Galeazzi, Department of Endodontics, Milan, Italy; ^5^ Silk Biomaterials srl, Lomazzo, Italy; ^6^ National Centre for Scientific Research “Demokritos”, Institute of Nanoscience and Nanotechnology, Aghia Paraskevi, Greece

**Keywords:** *Staphylococcus epidermidis*, implant-related bone infections, biofilm, nanoparticles, osteoconductive peptides, silk fibroin, local antibiotic delivery, prevention

## Abstract

**Introduction:**

Implant-related infections and infected fractures are significant burdens in orthopedics. *Staphylococcus epidermidis* is one of the main causes of bone infections related to biofilm formation upon implants. Current antibiotic prophylaxis/therapy is often inadequate to prevent biofilm formation and results in antibiotic resistance. The development of bioactive materials combining antimicrobial and osteoconductive properties offers great potential for the eradication of microorganisms and for the enhancement of bone deposition in the presence of infections. The purpose of this study is to prevent the development of methicillin-resistant *S. epidermidis* (MRSE)-infected nonunion in a rat model.

**Methods:**

To this end, a recently developed in our laboratories bioactive material consisting of antibiotic-loaded nanoparticles based on carboxylic acid functionalized hyperbranched aliphatic polyester (CHAP) that are integrated into peptide-enriched silk fibroin sponges with osteoconductive properties (AFN-PSF) was employed, whose biocompatibility and microbiological tests provided proof of its potential for the treatment of both orthopedic and dental infections. In particular, non-critical femoral fractures fixed with plates and screws were performed in Wistar rats, which were then randomly divided into three groups: 1) the sham control (no infection, no treatment); 2) the control group, infected with MRSE and treated with peptide-enriched silk fibroin sponges incorporating non-drug-loaded functionalized nanoparticles (PSF); 3) the treated group, infected with MRSE and treated with peptide-enriched silk fibroin sponges incorporating vancomycin-loaded functionalized nanoparticles (AFN-PSF). After 8 weeks, bone healing and osteomyelitis were clinically assessed and evaluated by micro-CT, microbiological and histological analyses.

**Results:**

The sham group showed no signs of infection and complete bone healing. The PSF group failed to repair the infected fracture, displaying 75% of altered bone healing and severe signs of osteomyelitis. The AFN-PSF treated group reached 70% of fracture healing in the absence of signs of osteomyelitis, such as abscesses in the cortical and intraosseous compartments and bone necrosis with sequestra.

**Discussion:**

AFN-PSF sponges have proven effective in preventing the development of infected nonunion *in vivo*. The proposed nanotechnology for local administration of antibiotics can have a significant impact on patient health in the case of orthopedic infections.

## 1 Introduction

Nonunion of infected long bones is still a clinical challenge posing outstanding problems in infection control with several difficulties in their management. Open fractures are definitely at greater risk for non-union. The rate of development of posttraumatic infection ranges from 1% to 2% of closed fractures and more than 30% of open fractures of the Gustilo-Anderson type III tibia (Walter et al., 2021). In general, orthopedic implants, such as fracture fixation devices and metal prostheses, exhibit the risk for the development of bone infections and osteomyelitis (Alt, 2017).

Staphylococci are the most common pathogens implicated in orthopaedic infections, resulting in prolonged hospitalization and long-term antibiotic therapy increasing the risk of multidrug resistance development. *S. epidermidis* is a low-virulence commensal of the human skin and it is one of the leading causes of infections associated with biofilm formation upon implants because of its remarkable ability to adhere and colonize medical devices. The biofilm formed by *S. epidermidis* makes the antibiotic treatment less effective and contributes to the development of antibiotic-resistant strains, such as methicillin-resistant *S. epidermidis* (MRSE), at a higher rate compared to other non-resistant staphylococcal species (i.e. *S. aureus*) (Shams and Rapp, 2004). Furthermore, *S. epidermidis*-mediated infections represent a complicated burden, being associated with a worse eradication rate, thereby negatively affecting the quality of life of elderly patients where antibiotic therapy acts poorly (Namvar et al., 2014; Hischebeth et al., 2019).

A MRSE clinical strain (Bottagisio et al., 2017) and animal models of nonunion fractures induced by MRSE have been developed to study pathogenesis and new preventive and therapeutic strategies (Lovati et al., 2016a; Lovati et al., 2016b; Lovati et al., 2018). Indeed, laboratory animal models of osteomyelitis are helpful in quantifying the ability of antimicrobial compounds to eradicate infections and facilitate bone growth.

Currently, the standard therapy for infected nonunion is based on the systemic administration of antibiotics in combination with an extensive surgical bone debridement to remove necrotic tissue, and the final implantation of bone substitutes (autografts or allografts) and implant devices (Reiter et al., 2013). To date, no method has generally been recognized as the gold standard method for treating osteomyelitis (Suchý et al., 2016). Thus, it can be especially difficult to balance the control of infection while ensuring the stability of the fracture healing in the presence of bone defect and osteomyelitis.

In this clinical context, two major issues must be addressed: the loss of bone tissue and the development of antibiotic resistance (Sahal and Bilkay, 2014). Thus, besides treating bone infection, it is mandatory to reestablish bony bridging to support the fracture stability. Although current bone substitutes permit the functional restoration of the damaged tissue, donor-site morbidity on one side, and immunological disorders on the other are the main drawbacks associated with the use of autografts and allografts, respectively. Therefore, there is an increasing demand for new biomaterials that can improve bone healing while delivering antimicrobials and providing targeted treatments to prevent bacterial bone infections (ter Boo et al., 2015; Metsemakers et al., 2015; Romanò et al., 2019).

Based on these premises, the incorporation of drug nanocarriers into bone-enhancing biomaterials - such as silk fibroin - seems a promising approach to cope with bone infections. Silk fibroins are fibrous proteins, produced by many insects and arachnids that have been widely used through history mainly for their outstanding mechanical properties.

In more recent years, the interest in the fibroin produced by domesticated silkworm (*Bombyx Mori*), grew also in biomedical sciences field thanks to its inherent biocompatibility (Panilaitis et al., 2003; Meinel et al., 2005; Leal-Egaña and Scheibel, 2010) its favorable degradation mechanism that leads to nontoxic products (Horan et al., 2005; Wang et al., 2008; Biggi et al., 2020) and the possibility to modify the material properties to suit a wide range of applications. Moreover, it has been already approved by FDA as biomaterial for tissue engineering. Despite lacking an inherent antimicrobial activity, Silk Fibroin (SF) is highly suitable for the preparations of carriers for the delivery of sensible drugs. Such molecules can be included in the scaffold without the need of harsh conditions or organic solvents and subsequently efficiently protected from environment thanks to Silk Fibroin peculiar properties (Griffanti et al., 2018; Lovati et al., 2020).

In order to further improve the performance of SF-based materials, nanotechnological approaches have been applied, providing nanobiomaterials with specific properties, such as antibacterial or controlled release (Merino et al., 2015; Xu et al., 2019). Various nanoparticles - ceramics (calcium carbonate, calcium phosphate), inorganic (silicates, halloysite nanotubes, etc.), metal/metal-oxide (silver, gold, iron oxide, etc.), carbon-based (graphene oxide, carbon nanotubes, etc.) and polymeric (chitosan, alginate, dendritic polymers, etc.) - have been integrated into SF matrices, offering nanobiocomposites with targeted properties needed in biomedical fields (Yan et al., 2014; Pan et al., 2015; Patil and Singh, 2019; Karamat-Ullah et al., 2021; Eivazzadeh-Keihan et al., 2022; Motasadizadeh et al., 2022). Furthermore, these nanoparticles are known to have been extensively studied as drug delivery systems (Nikezić et al., 2020).

Among these, the family of dendritic polymers - highly branched macromolecules with nanosized dimensions consisting of repeating units and a large number of terminal functional groups - is one of the most studied categories of drug delivery systems (Kurniasih et al., 2015; Paleos et al., 2016; Paleos et al., 2017; Sideratou et al., 2018; Cook and Perrier, 2019; Wang et al., 2022). Due to their three-dimensional architecture, nanocavities can efficiently encapsulate various bioactive compounds such as anticancer drugs, antibiotic drugs, etc. to improve solubility, enhance their activity and reduce their severe side effects. Moreover, due to their terminal functional groups, they can be easily functionalized, affording functional nanocarriers with low toxicity, high loading capability, controlled release properties or other advantageous properties such as antibacterial properties (Paleos et al., 2007; Paleos et al., 2008; Hou et al., 2009; Cook and Perrier, 2019; Duan et al., 2022).

In our recent study, a series of peptide-enriched silk fibroin sponges, augmented with antibiotic-loaded nanoparticles, that are based on a partially carboxylated hyperbranched aliphatic polyester, were prepared and assessed *in vitro* against a group of bacteria related to orthopedic or dental implant-related infections (Sideratou et al., 2022). It was found that among the different formulations tested, the peptide-enriched silk fibroin sponges containing vancomycin-loaded functionalized polyester nanoparticles were the safest and most effective for the extirpation of bacteria related to orthopedic or dental infections compared to the other tested antibiotic-loaded sponges.

Encouraged by these results, in the present study, we verified the hypotheses that peptide-enriched silk fibroin sponges loaded with antibiotic functionalized nanoparticles (AFN-PSF) could decrease the rate of bone infection and increase new bone growth in a rat model of MRSE-infected femoral fracture as compared to the same peptide-enriched silk fibroin (PSF) without antibiotic or untreated non-infected, non-critical size fractures. To our knowledge, no such comprehensive *in vivo* evaluation has been conducted to date with respect to a combination of osteoinductive silk fibroin-based materials and nanoencapsulated antibiotics to treat bone infections.

## 2 Materials and methods

### 2.1 Development of peptide-enriched silk fibroin sponges loaded with vancomycin-loaded functionalized nanoparticles (AFN-PSF)

Peptide-enriched silk fibroin sponges (PSF) loaded with vancomycin-loaded functionalized nanoparticles were prepared according to the methodology described in our previous publications (Sideratou et al., 2022). In brief, silk cocoons were degummed in deionized water to remove sericin. The obtained pure silk fibroin fibers were dissolved in 9.3 M LiBr solution, which was then dialyzed to obtain the final solution at a concentration of 3.3% w/v. Fibroin-derived anionic polypeptides (Cs) were prepared by treating an aqueous silk fibroin solution with α-chymotrypsin and obtained after freeze-drying as previously described (Marelli et al., 2012).

**Figure 1:**
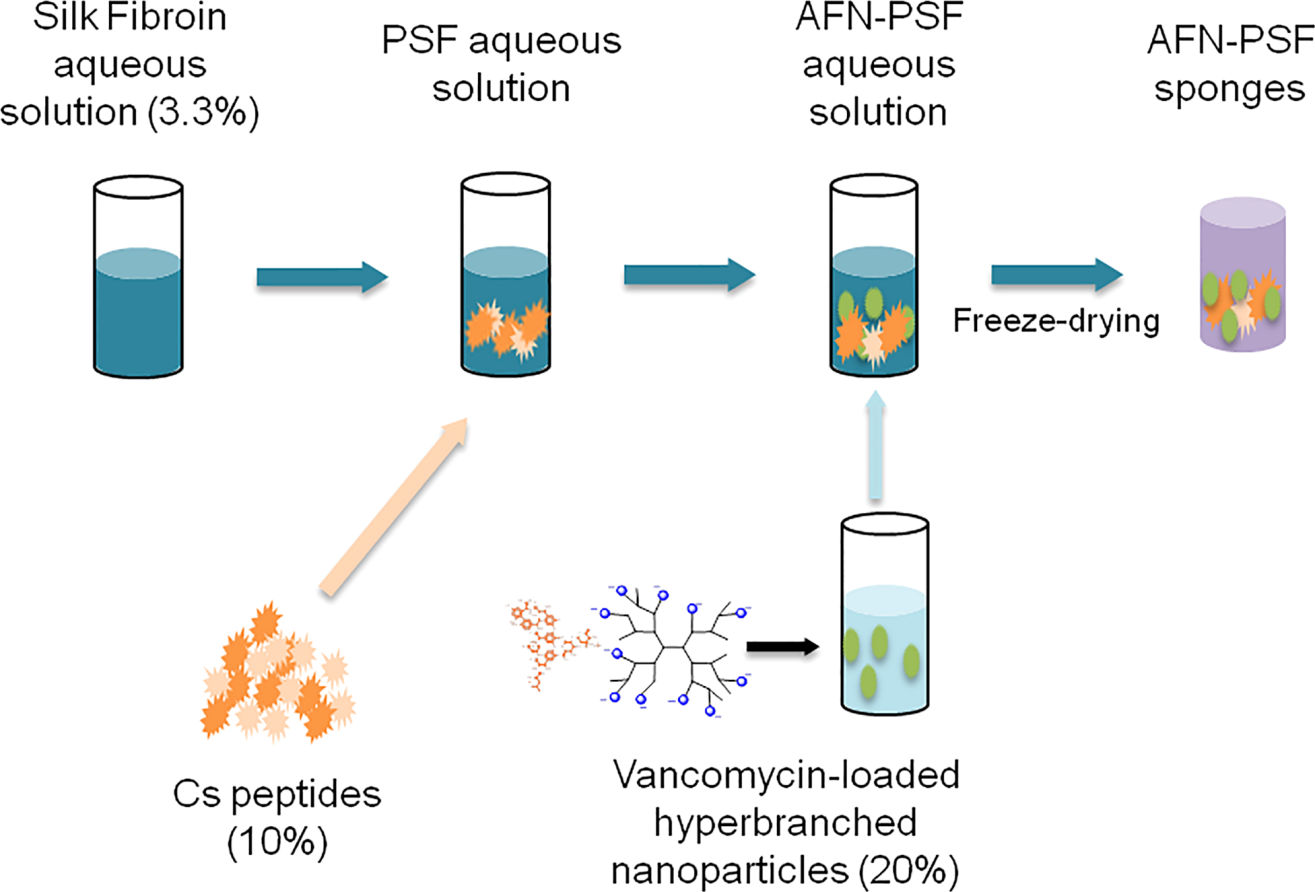
Schematic illustration of the development of peptide-enriched silk fibroin sponges loaded with vancomycin-loaded functionalized nanoparticles (AFN-PSF).

Partially carboxylated hyperbranched aliphatic polyesters with 20 carboxyl groups (CHAP) based on commercially available BoltornTM H40 (BH40) were prepared by the reaction of the hydroxyl end groups of BH40 with succinic anhydride, in anhydrous basic conditions (Bode et al., 2017). Subsequently, this negatively charged polymeric derivative interacted in aqueous media with positively charged vancomycin through electrostatic interactions as well as through hydrogen bonds, leading to the formation of ~80 nm diameter nanoparticles (CHAP_VC) with a zeta potential value of –39.5 mV and vancomycin loading of 39.5% w/w. Subsequently, peptide-enriched silk fibroin sponges containing either vancomycin-loaded functionalized CHAP nanoparticles (AFN-PSF) or non-drug-loaded CHAP (PSF) were prepared by enriching a silk fibroin with 10% w_Cs_/w_SF_ Cs peptides and 20% w_AFNs_/w_SF_ CHAP_VC or CHAP as detailed elsewhere (Sideratou et al., 2022). The schematic illustration of the biomaterial design is reported in **Figure 1**.

### 2.2 *In vitro* evaluations of AFN-PSF sponges

The antibacterial properties and the ability of the antibiotic-loaded silk fibroin products to select the tested bacterial strains for antibiotic resistance were extensively investigated in a recently published study (Sideratou et al., 2022).

In particular, the bactericidal activity of AFN-PSF sponges was tested by means of killing curves over time, to verify that the antibacterial load and release was sufficient to determine a 3-log reduction in CFU/mL (99.9% kill) from the initial inoculum.

Furthermore, to exclude the accidental selection of antibiotic resistant strain due to the selective pressure of the antibiotic released by AFN PSF sponges tested clinical isolates were subcultured in the presence of the antibiotic-loaded silk fibroin products. Briefly, bacteria were cultured in the presence of AFN PSF sponges over a 7-day period and, subsequently subcultured for another 7 days to exclude any transient or stable reduced susceptibility to antibiotics.

Lastly, to exclude any cytotoxic activity against eukaryotic cells in view of *in vivo* testing, the potential cytotoxic effect of PSF sponges either functionalized with antibiotics or not was investigated.

More information on the aforementioned *in vitro* evaluations preparatory to the following *in vivo* testing can be found in the article recently published by our research group (2022).

### 2.3 Ethic statement and study design

The animal study was approved by the Animal Care and Use Committee (IACUC) of the Mario Negri Institute for Pharmacological Research (IRFMN) (Permit number 557/2021-PR). The rats were managed in accordance with EU legislation (Council of the EC Directive 2010/63/EU) and Italian law (D. legs 26/2014). Animal health and well-being, protocols and experimental procedures were systematically verified by a certified veterinarian. All surgical procedures were carried out under general anesthesia and every effort was made to minimize animal suffering. The study consisted of twenty-seven 12-week-old male Wistar rats (body weight 374 ± 32 g) (Charles River Laboratories SRL, Calco, Lecco, Italy). The rats were randomly divided into three groups: 1) the sham control, no infection, no treatment (SHAM) (n = 7); 2) the control group infected with bacteria and treated with peptide-enriched silk fibroin loaded with CHAP (PSF) (n = 10); and 3) the treated group infected with bacteria and treated with antibiotic (vancomycin)-loaded CHAP nanoparticles in PSF (AFN-PSF) (n = 10).

### 2.4 *In vivo* procedures

**Figure 2:**
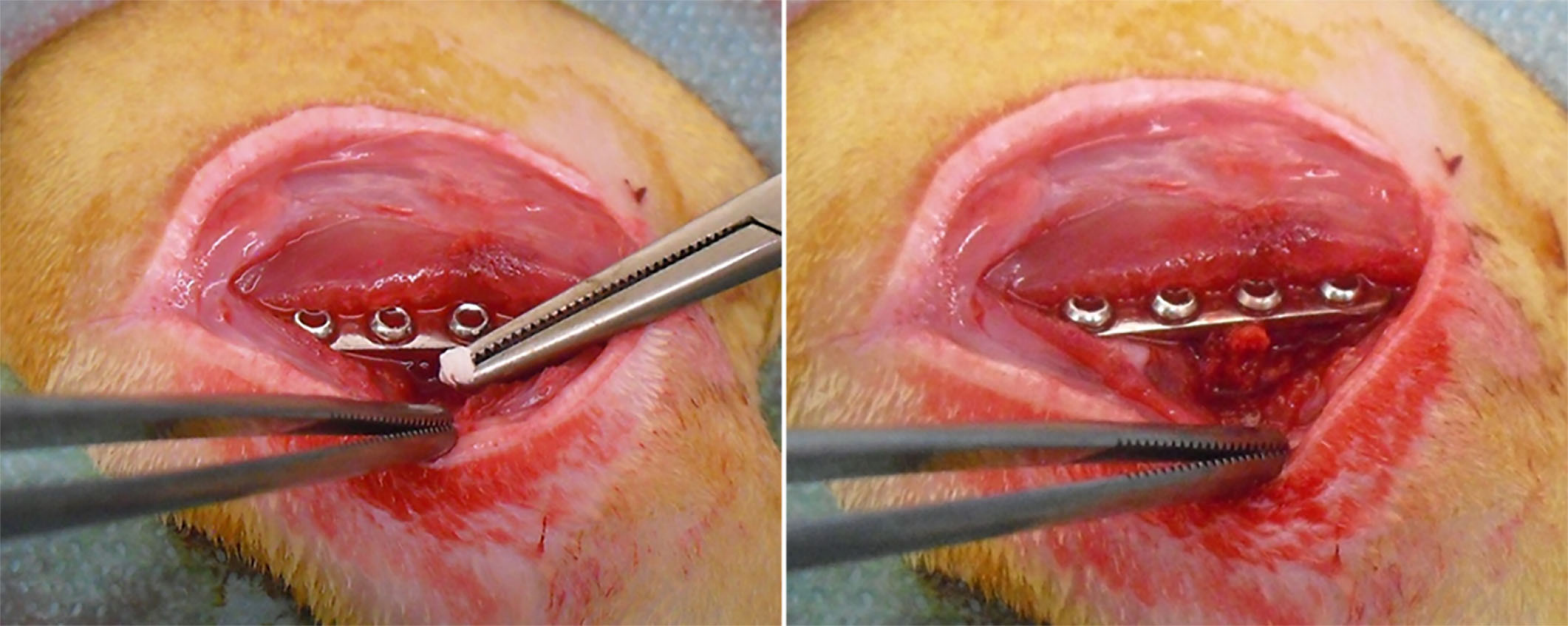
AFN-PSF treatment. Vancomycin-loaded functionalized PSF sponge (AFN-PSF) is placed into the fracture site after the fracture osteosynthesis.

As previously described in other studies (Lovati et al., 2016a; Lovati et al., 2016b), under general anesthesia, the osteotomy of the right femur was performed in all rats, then synthesized with stainless steel plate and bicortical screws (all from Zimmer, Germany). Immediately after the osteosynthesis, the non-critical femoral defect (1 mm) was injected with 30 µL of sterile saline solution in the SHAM group, or an inoculum of 1×10^5^ CFU/30 µL of MRSE strain #GOI1153754-03-14 (Bottagisio et al., 2017) in PSF and AFN-PSF groups, according to the already developed animal model (Lovati et al., 2016a). Thereafter, the SHAM group did not receive any further treatment, while 6 mg of unloaded PSF sponge (PSF group) or vancomycin-loaded PSF sponge (AFN-PSF group) were placed into the fracture site (**Figure 2**). Finally, the muscle and skin layers were closed by Vicryl 4/0 and Ethilon 4/0 (Ethicon, Johnson&Johnson), respectively. Perioperative, all rats received cefazolin (30 mg/kg IM, Cefazil, Italfarmaco) and carprofen (5 mg/kg SC, Rimadyl, Pfizer). After surgery, buprenorphine (0.1 mg/kg SC, Temgesic, Schering Plough) and atipamezole (1 mg/kg SC, Antisedan, Pfizer) were administered. Animals were monitored daily for their overall condition and well-being, clinical signs of infection, lameness, weight-bearing, swelling, local hyperemia, wound healing, serous exudate, hematoma, pain, and suffering. Changes in body weight and the number of white blood cells were monitored on a weekly basis. Eight weeks later, the rats were euthanized with CO_2_. Micro-CT scans, histological analyses, and microbiological tests were conducted on explanted limbs to assess bone healing and infection.

### 2.5 Body weight and blood analyses

Body weight (b.w.) was measured on the day of surgery (D0) and weekly up to the day of explantation (D56) and reported as relative b.w. increase on the D0 baseline. On days 0, 7, 14, 42 and 56, venous blood was harvested from the tail vein under general anesthesia and then transferred into K_2_EDTA tubes (Microtainer MAP, Becton Dickinson) to count white blood cells.

### 2.6 Micro-CT analysis and measurement

The micro-CT images of each femur (n= 5 SHAM; n = 8 PSF; n = 10 AFN-PSF) were obtained using a Skyscan 1076 micro-CT device (Skyscan, Aartselaar, Belgium). The scanning parameters were set at 100 kV, 100 mA, exposure time 1000 ms, filter Al 0.5, rotation step 0.5 deg, and a voxel resolution of 9 µm. Volume images were reconstructed using NRecon software (Skyscan) at a pixel size of 9 μm with 256 gray levels. The reconstructed volume was read by DataViewer software (Skyscan) to align and obtain a volume of interest (VOI). The obtained VOI was read by CTAn software (Skyscan) to measure the percentage of bony bridging of the fracture gap (>75%, 50–75%, or <75%) and the bone volume (BV) calculated within the volume of interest (BV/TV%), as described elsewhere (O'Neill et al., 2012).

### 2.7 Microbiological analysis and evaluation of antibiotic-resistant selection

After 56 days, tissues were collected by standard sterile technique. Briefly, femurs were harvested immediately after sacrifice, and bacteria were recovered from explanted femurs (n = 4 PSF and n = 5 AFN-PSF) through the sonication of the samples in 0.1% w/v dithiothreitol (DTT) solution to dislodge bacteria from tissues, plates, and screws. The DTT eluate was then centrifuged at 3000 rpm for 10 min at 4°C, and the bacterial pellet was resuspended in 1 mL of sterile saline solution. Lastly, 100 μL of the sample was plated on Mannitol Salt Agar (MSA) and Tryptic Soy Agar (TSA), and plates were incubated at 37°C for 24 hours. Viable bacterial colonies were counted and data were reported as CFU/mL. The limit of detection was set at 1 x 10^2^ CFU/mL. The antibiotic resistance profile of MRSE prior to and following *in vivo* experiments was investigated by means of the Vitek2 System (BioMeriéux).

### 2.8 Histological analyses

The femurs (n = 4 PSF; n = 5 SHAM and AFN-PSF) were fixed in 10% formalin for 4 days, decalcified with 14% EDTA for 20 days. The decalcification endpoint was evaluated by bending and probing (needle puncture) the samples. Then, the samples were embedded in paraffin and cut into 5 µm sections. Haematoxylin and eosin (H&E) staining was performed to assess morphology, inflammation, fracture healing, and signs of osteomyelitis. Additional sections underwent modified Brown & Brenn staining (Taylor, 1966) to confirm the presence or absence of bacteria. Digital slides were obtained by using the NanoZoomer S60 Digital slide scanner (Hamamatsu, C13210-01), and images were captured by using the NDP.view2 Viewing software (Hamamatsu, U12388-01). To evaluate the signs of osteomyelitis of the periosteum, cortex and medullary canal, two different scores were applied, namely: the total score based on the human Petty scale (0-3) (Petty et al., 1985; Lovati et al., 2013) and a more suitable score for the rat species developed by the veterinary histopathologist in a range of 0-5: 0, normal bone; 1, complete bony bridging w/o inflammation, not to slight periosteal reaction, not to slight fibrovascular tissue; 2, complete bony bridging with minimal to slight inflammation, minimal to slight periosteal reaction and minimal to slight fibrovascular tissue; 3, absence of bony bridging, minimal to slight inflammation, slight to moderate fibrovascular tissue +/- cartilaginous metaplasia; 4, absence of bony bridging, slight to moderate inflammation, moderate to marked fibrovascular tissue +/- cartilaginous metaplasia; and 5, absence of bony bridging, marked to severe inflammation, marked to severe fibrovascular tissue +/- cartilaginous metaplasia.

### 2.9 Statistical analysis

The sample size was calculated on the basis of a previous study (Lovati et al., 2016a) through a paired t-test with α error = 0.05% and 80% power (G*Power 3.1 software, Düsseldorf, Germany) (Faul et al., 2007). Statistical analyses were carried out with GraphPad Prism 5 software (GraphPad Software, San Diego, California, USA). After checking the normal distribution of the data using the Shapiro-Wilk test, the inter-group comparisons were analyzed with one-way analysis of variance (ANOVA) and then coupled with Bonferroni’s *post hoc* test. All data are reported as means ± standard deviation (SD). The level of significance was p < 0.05.

## 3 Results

### 3.1 Clinical examination

Four animals (n = 2 SHAM and n = 2 PSF) died within 1 hour of surgery from anesthetic complications. Thus, the remaining rats per group were 5, 8 and 10 for the SHAM, PSF and AFN-PSF groups, respectively. During the monitoring period, no additional animals died. Two rats of the PSF and two of the AFN-PSF group showed a local swelling around the fracture site, but a complete load bearing between day 7 and 21 after the bacterial inocula. No other local (erythema) or systemic (fever or lethargy) clinical signs of infection were observed.

### 3.2 Body weight and blood analyses

**Figure 3:**
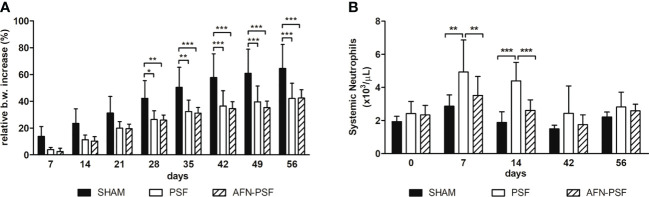
Clinical data. **(A)** The histogram depicts the relative body weight increase in the experimental groups over time. **(B)** The histogram depicts the systemic neutrophil count in the experimental groups over time. Comparisons between groups and time points were analyzed with two-way ANOVA and Bonferroni’s *post hoc* test. Statistical significance was p < 0.05 (*), < 0.01 (**), and < 0.001 (***); n = 5 SHAM, n = 8 PSF, n = 10 AFN-PSF.

The relative increase in body weight from the baseline (D0) is indicated in **Figure 3A**. All rats in the SHAM group showed a gradual increase of body weight over time, consistent with their growth curve, without significant differences with the other experimental groups. Otherwise, from day 28 onwards, the PSF and AFN-PSF rats recorded a slower body weight increase than the SHAM group with a statistical difference. In **Figure 3B**, the neutrophil count is reported as number of cells×10^3^/µL. On days 7 and 14, the PSF rats exhibited a significant neutrophil increase compared to both the SHAM and AFN-PSF groups. After 42 days, the blood values normalized in all groups with no significant differences.

### 3.3 Micro-CT imaging

**Figure 4:**
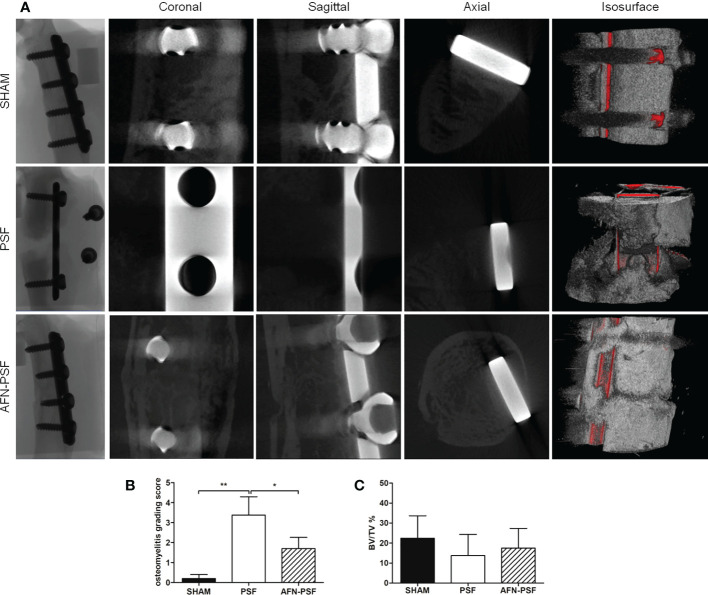
Qualitative micro-CT imaging, isosurface, and quantitative analyses. **(A)** The representative panel shows micro-CT images on the day of explantation at the sagittal, coronal, and axial planes. 3D isosurface reconstruction is also depicted. **(B)** Osteomyelitis grading score based on Odekerken’s scale is reported in the histogram in panel. **(C)** Quantitative BV/TV % is reported in panel. Comparisons among groups were analyzed with one-way ANOVA corrected with Bonferroni’s *post hoc* test. Statistical significance was p < 0.05 (*) and < 0.01 (**); n = 5 SHAM, n = 8 PSF, n = 10 AFN-PSF.

Micro-CT analysis revealed a different percentage of bony bridging in the experimental groups, as reported in **Table 1** (n = 5 SHAM, n = 8 PSF, n = 10 AFN-PSF). Specifically, the SHAM group showed a complete bony bridging in most of the cases (80%) and only in one rat (20%) a partial closure of the fracture and a mild cortical reaction was found even if in the absence of signs of osteomyelitis. In the PSF group, a higher percentage of nonunion or partial healing of fractures (75%) was measured than in the AFN-PSF group. In particular, the PSF group describes an evident nonunion associated with severe osteolysis, loss of screw stability, femoral diaphysis deformation and dislocation of bone stumps. In the AFN-PSF group, only 40% of the samples showed a poor bony bridging related to mild signs of osteomyelitis, while 60% showed a complete closure of the fracture site with well-structured bone callus and mineralized cortices in the absence of mild or severe signs of osteomyelitis. In **Figure 4**, the qualitative micro-CT analysis (**Figure 4A**), the grading score (**Figure 4B**) and the quantitative analysis (**Figure 4C**) are reported.The osteomyelitis grading score according to the Odekerken’s scale (Odekerken et al., 2013) supported the clinical data showing a significant difference between the PSF and both the SHAM (p<0.01) and PSF-AFN groups (p<0.05), while no differences were found between SHAM and AFN-PSF groups (**Figure 4B**). Despite the fact that no significant differences among groups were found in the quantitative analysis of BV/TV (%) (**Figure 4C**), the overall trend showed higher values of AFN-PSF samples compared to PSF even though lower with respect to the SHAM group.

**Table 1:** Percentage of bony bridging of the fracture site.

Groups	Bony bridging <50% Nonunion fracture	Bony bridging 50-75% Partial fracture healing	Bony bridging >75% Fracture healing
SHAM	0% (0/5)	20% (1/5)	80% (4/5)
PSF	62,5% (5/8)	12,5% (1/8)	25% (2/8)
AFN-PSF	30% (3/10)	10% (1/10)	60% (6/10)

### 3.4 Microbiology

After 24 hours of culture on MSA plates, mannitol negative, white/smooth colonies were counted and the number of CFU/mL recorded. The 50% of explanted samples belonging to the PSF group resulted in a positive microbiological culture: in 2 specimens out of 4, 2.43 x 10^2^ and 43 CFU/mL were counted. Whereas only from 1 sample out of 5 belonging to the AFN-PSF group, viable colonies were retrieved (1.66 x 10^2^ CFU/mL). None of cultures taken from contralateral limbs (negative controls) were found positive. The identification of the coagulase-negative colonies from the microbiological cultures was then confirmed by the Vitek2 system (BioMeriéux), along with the definition of their antibiotic resistance profile. No differences in the antimicrobial resistance profile were detected between the parental bacterial strain and strains retrieved after the *in vivo* experimentation. In particular, all strains were susceptible to gentamicin, vancomycin and fusidic acid (MIC ≤ 0.5μg/mL), erythromycin and daptomycin (MIC ≤ 0.25μg/mL), clindamycin and tigecycline (MIC ≤ 0.12μg/mL), linezolid and tetracycline (MIC ≤ 1μg/mL), teicoplanin (MIC=4μg/mL), trimethoprim/sulfamethoxazole (MIC ≤ 10μg/mL), while they were resistant to benzylpenicillin (MIC≥0.5μg/mL), oxacillin, cefazolin, rifampicin and levofloxacin (MIC≥4μg/mL).

### 3.5 Histology

**Figure 5:**
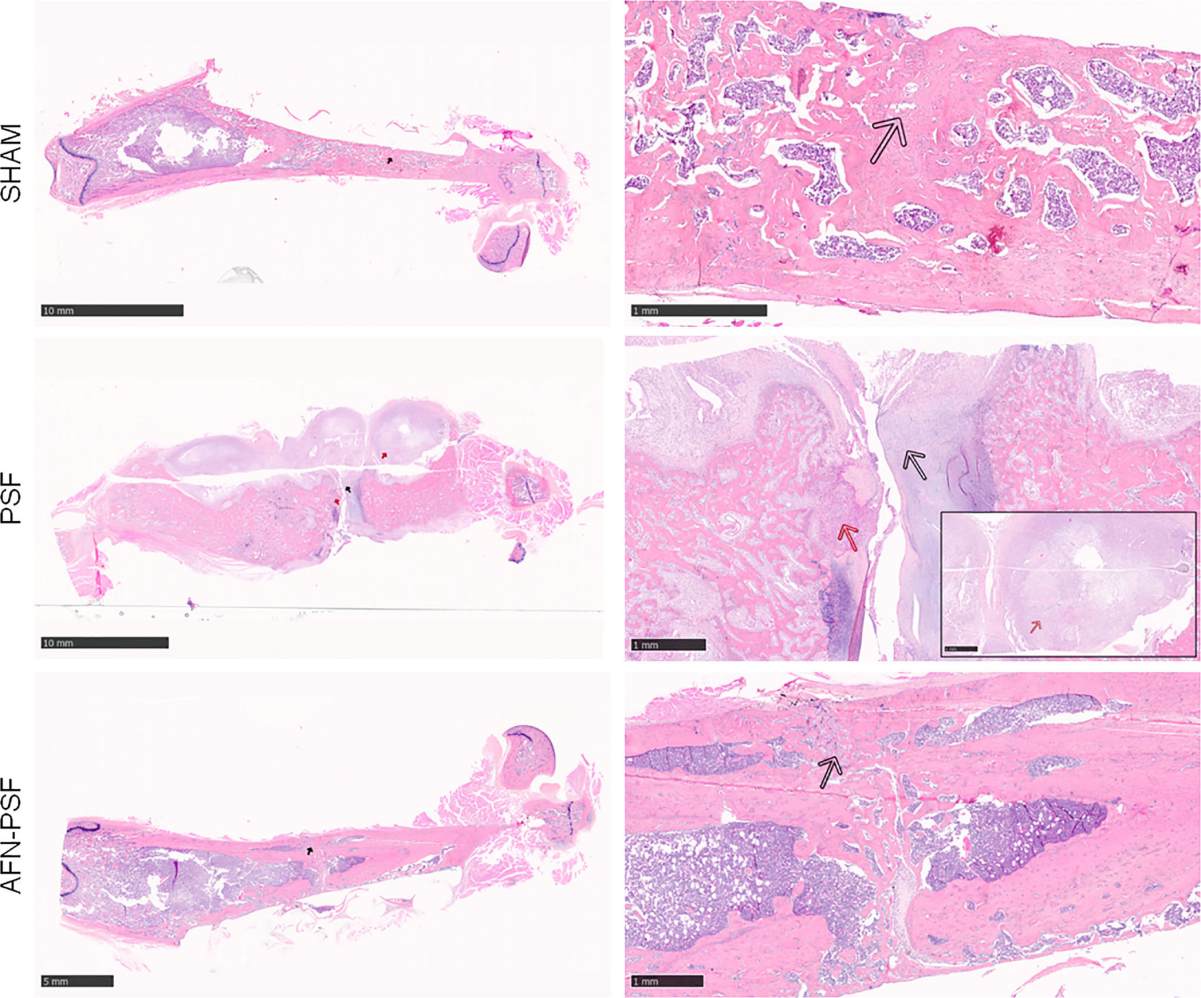
Qualitative H&E histological panel. The representative panel shows the complete fracture healing with new bone formation in a remodeling phase (black arrow) of the SHAM and AFN-PSF groups. The PSF group showed a complete disorganization of the bone structure and abundant fibrovascular tissue typical of nonunion (black arrow), as well as the presence of chronic abscesses and necrotic tissue (red arrows). Scale bars at 10 mm (left side) and 1 mm (right side).

In **Figure 5**, a representative panel of histopathological analysis is reported.

The SHAM group showed a complete fracture healing in most of the samples (4/5) associated with new bone formation in a remodeling phase and newly-formed bone covering the implant. A single sample exhibited a partial fracture healing closed by fibrovascular tissue and hypertrophic/hyperplasic cartilaginous tissue. No evidence of osteomyelitis was detected as expected.

The PSF group most often (4/5) showed a complete disorganization of the bone structure and absence of bony bridging and abundant fibrovascular tissue typical of nonunion. The cortices appeared uniformly enlarged with rare areas of bone remodeling. A single specimen depicts moderate early intraosseous fibrosis. All samples presented chronic abscesses, necrotic tissue and marked to severe inflammation at the periosteal level and extending to the intraosseous level, with the presence of macrophages and granulocytes, and granulation tissue. The absence of evident bacteria (Brown & Brenn staining) in the specimens explanted after 8 weeks confirmed the variability of results obtained in the microbiological analyses.

**Figure 6:**
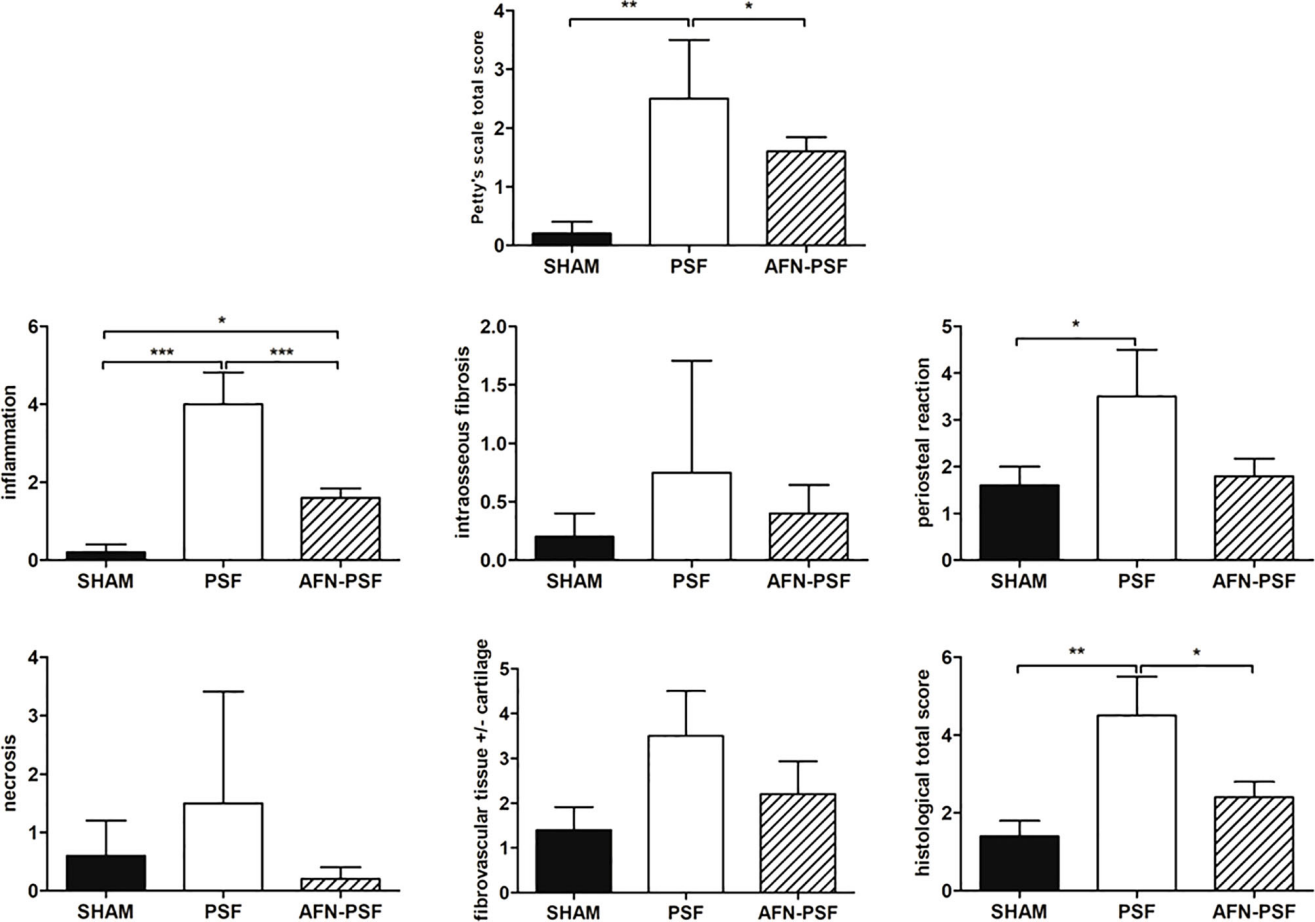
Semi quantitative histological score. The grading scores are reported in the histograms as human-based Petty’s scale for osteomyelitis signs in periosteum, cortex and medullary canal, as well as for inflammation, intraosseous fibrosis, periosteal reaction, bone necrosis, fibrovascular tissue +/- cartilaginous tissue, and total score based on a rat-specific scale. Comparisons among groups were analyzed with one-way ANOVA corrected with Bonferroni’s *post hoc* test. Statistical significance was p < 0.05 (*), < 0.01 (**), and < 0.001 (***); n = 5 SHAM, n = 4 PSF, n = 5 AFN-PSF.

The AFN-PSF group showed almost complete to complete fracture healing in most of the samples (4/5) associated with new bone formation in a remodeling phase and neoformed bone covering the implant, even though a slight to moderate reactive periosteum was detected in all the samples, with some dispersed inflammatory cells. Only one case presented the absence of bony bridging with a nonunion establishment associated with abundant fibrovascular tissue and cartilaginous metaplasia, but in the presence of a minimal inflammation and no signs of osteomyelitis. The semi quantitative score was performed on a rat-specific scale from 0 (absent), 1 (minimal), 2 (slight), 3 (moderate), 4 (marked) to 5 (severe) to describe the inflammation, periosteal reaction, fibrovascular tissue +/- cartilaginous tissue, intraosseous fibrosis, and bone necrosis. The samples were also analyzed on the basis of the human-specific Petty scale already used elsewhere that confirmed the statistical data. The histograms in **Figure 6** show a clear trend among the three groups analyzed, in which the PSF group still displays values in grades marked to severe. In particular, the PSF group depicted a very significant difference in terms of inflammation with respect to both the SHAM and AFN-PSF groups (p<0.001). The inflammatory profile was also significant in the AFN-PSF group in comparison with SHAM (p<0.05). The total score, as the analysis of the overall histological appearance of the samples, maintained the trend where PSF group shows a significant difference compared to SHAM and AFN-PSF for p<0.01 and p<0.05, respectively.

## 4 Discussion

Osteomyelitis is a frequent infectious disease of bone that requires apposite management. When it comes to osteomyelitis, the bacteria of most concern are *Staphylococcus aureus* and *epidermidis*. The conventional approach of implant-related bone infections is a lingering systemic administration of antibiotics, often associated with a revision surgery to remove the infected implants and surrounding tissues, thus leading to bone defects (Høiby et al., 2015). The main drawbacks of the use of systemic antibiotics are the potential toxicity and the development of microbial resistance. Ideally, the method of administration should allow the antibiotic to release locally in a strong early concentration. Next, a lower concentration, but still effective over a long period of time (days) is still required, thus effectively preventing bacterial growth (Gogia et al., 2009; McLaren et al., 2014).

As the ultimate advance for the treatment of infected bone is to simultaneously repair large-size bone defects and to inhibit related infections, the use of an osteoconductive bone material with antibiotic release capabilities paired with osteogenesis is possible (Mao et al., 2015).

With the purpose to deliver antibacterial drugs, nanotechnology has gained space in the recent years. In this field, antibiotic-loaded nanomaterials are valuable to improve antimicrobial activity in low doses and to maximize the long-term release of antibiotics, thus decreasing the development of antimicrobial resistance (Sharma et al., 2012; Kalhapure et al., 2015). In this context, functional dendritic nanocarriers with low toxicity, high charge capacity and controlled release properties may be suitable candidates (Paleos et al., 2007; Paleos et al., 2008; Stagni et al., 2020; Duan et al., 2022). However, a few of antimicrobial-based nanotechnologies able to treat infectious biofilm is actually translated from the bench to the bedside (Liu et al., 2019).

In our recent study, we developed, screened and optimized osteoconductive peptide-enriched silk fibroin (PSF) sponges incorporating antibiotic-loaded functionalized nanoparticles (AFN) that are based on carboxylated hyperbranched aliphatic polyesters, by analyzing antibiotic release kinetics, antimicrobial efficiency and cytocompatibility (Sideratou et al., 2022). The biocompatibility and microbiological tests revealed that, among the various tested formulations, vancomycin-loaded PSF-ANF sponges were the safest and most effective products for the eradication of *in vitro* orthopedic related bacterial contamination.

Thus, in the present work, the above mentioned vancomycin-loaded nanobiocomposite sponges were used for the local treatment of MRSE-induced nonunion employing a well-characterized rat model effective in developing osteomyelitis and septic nonunion (Lovati et al., 2016a). Our purpose is to *in vivo* evaluate this silk fibroin-based nanobiocomposite loaded with vancomycin that will not only accomplish the antimicrobial goal, but also may promote bone growth following the elimination of infection by including osteoconductive peptides into the delivery systems.

On days 7 and 14, the PSF rats exhibited a significant neutrophil increase compared to both the SHAM and AFN-PSF groups, thus demonstrating that in the presence of bacteria, but in the absence of a local antimicrobial treatment, there was a significant immune response of the host against infections. This data is consistent with our previous study in which rats infected with the same bacterial strain and left untreated (positive control, PC) had an abnormal high level of neutrophils similar to the PSF group (Lovati et al., 2016b). Consequently, also the osteomyelitis grading score based on the Odekerken’s scale in the PSF group was equal to the positive control group (Lovati et al., 2016b).

Here, we also demonstrated that, in the AFN-PSF group, the antibiotic-loaded nanobiocomposite satisfied the osteoconductive properties by occupying bone gaps and enhancing the osteogenesis, thus confirming bone deposition already assessed in an ovine model of bone gaps (Lovati et al., 2020). Moreover, the nanoparticles delivered vancomycin with adjustable release kinetics (Sideratou et al., 2022) able to eradicate bacteria, to prevent the prime step of biofilm formation and bacterial adherence (Masters et al., 2019), and to permit an efficient bony bridging. Indeed, both the osteomyelitis grading score and the percentage of bony bridging in the AFN-PSF treated group was higher than the use of vancomycin-enriched hydrogel layered on the plate surface before the fracture stabilization, as employed elsewhere (Lovati et al., 2020). This data support the hypothesis that nano-encapsulated antibiotics may have a powerful capability in fighting bacteria than antimicrobial drugs embedded in hydrogel or cement materials. More importantly, the semi quantitative histological score showed a significant difference between the AFN-PSF and the PSF group both in terms of total score and inflammatory patterns, in which PSF depicted higher values. An increase in the total score and bone inflammation contributes significantly to poor bone healing and osseodeposition (Zhou et al., 2018). In fact, bacterial biofilm and osteomyelitis have been shown to be responsible for the production of an acid environment. This environment eventually results in the dissolution of carbonates and calcium ions, thus inhibiting bone healing (Esmonde-White et al., 2013).

The presence of persistent infection in PSF group appeared to have a negative effect on bone growth, thus supporting data obtained by others in animal models treated with cement without impregnated antibiotics (Hak, 2007; Ferguson et al., 2019).

Furthermore, we previously evaluated *in vitro* that 55% of vancomycin were released from AFN-PSF at a stable rate in the first hour, and then slowly released over the next 3 days (Sideratou et al., 2022). This means that the critical moment of most relevant orthopedic surgeries could potentially be protected by the new bioactive nanomaterial (Nast et al., 2016; Nichol et al., 2021), as also demonstrated in the present *in vivo* study.

Although, this study is limited in the assessment of the local impact of vancomycin because the concentration of the released antibiotic has not been determined, the results demonstrated that AFN-PSF has a sufficient antimicrobial activity to prevent bone infection *in vivo*. This innovative antibiotic-loaded nanobiocomposite shows the clinical features for local prophylaxis in arthroplasty or osteosynthesis with a special focus on application in patients at high risk of infection (Stewart and Bjarnsholt, 2020).

## 5 Conclusion

This study demonstrated that the nanotechnology for local antibiotic delivery combined with the osteoconductive properties of peptide-enriched silk fibroin AFN-PSF sponges has the potential to prevent infection, while stimulating bone deposition in the presence of infected fractures synthesized with metal plates. The antimicrobial activity of AFN-PSF was found to be sufficient to prevent the development of bone infection *in vivo* as proved in the rat femoral fracture model with the inoculation of a MRSE strain.

## Data availability statement

The raw data supporting the conclusions of this article will be made available by the authors, without undue reservation.

## Ethics statement

The animal study was reviewed and approved by the Animal Care and Use Committee (IACUC) of the Mario Negri Institute for Pharmacological Research (IRFMN) (Permit number 557/2021-PR).

## Author contributions

MBo, ZS, DT and AL conceptualized and designed the research. MBo, MBi, ZS, DT and AL designed experiments and performed analysis. MBi, DT and ZS prepared samples for *in vivo* studies. SP, SL and AL performed the animal surgeries. MBo performed microbiological analyses. FS imaged femur explants using a micro-CT scanner. PS performed histological analyses. MBo and AL. interpreted the data and wrote the manuscript. All authors contributed to the article and approved the submitted version.
